# Mechanism of lnRNA-ICL involved in lung cancer development in COPD patients through modulating microRNA-19-3p/NKRF/NF-κB axis

**DOI:** 10.1186/s12935-023-02900-2

**Published:** 2023-04-03

**Authors:** Jingjing Lu, Yan Shi, Feng Zhang, Ying Zhang, Xiangwang Zhao, Haiyan Zheng, Lingyu Li, Shiqiao Zhao, Liming Zhao

**Affiliations:** 1 Department of Respiratory and Critical Care Medicine, Shanghai East Hospital, Tongji University School of Medicine, NO. 150 Jimo Road, Shanghai, 200120 China; 2 Institute for Clinical Trials of Drug, Shanghai East Hospital, Tongji University School of Medicine, Shanghai, 200120 China; 3 Department of Pharmacy, Changzheng Hospital, Naval Medical University, No. 415, Fengyang Road, Shanghai, 200003 China; 4 Department of Emergency Medicine, Shanghai East Hospital, Tongji University School of Medicine, NO. 150 Jimo Road, Shanghai, 200120 China; 5 Department of Biostatistics, Yale School of Public Health, New Haven, CT 06510 USA

**Keywords:** Inhibitor associated with COPD and lung cancer (ICL), Chronic obstructive pulmonary disease (COPD), Lung cancer (LC), Hsa-miR19-3p, NF-κB, NKRF

## Abstract

The incidence of lung cancer (LC) in chronic obstructive pulmonary disease (COPD) patients is dozens of times higher than that in patients without COPD. Elevated activity of nuclear factor-k-gene binding (NF-κB) was found in lung tissue of patients with COPD, and the continuous activation of NF-κB is observed in both malignant transformation and tumor progression of LC, suggesting that NF-κB and its regulators may play a key role in the progression of LC in COPD patients. Here, we report for the first time that a key long non-coding RNA (lncRNA)-ICL involved in the regulation of NF-κB activity in LC tissues of COPD patients. The analyses showed that the expression of ICL significantly decreased in LC tissues of LC patients with COPD than that in LC tissues of LC patients without COPD. Functional experiments in vitro showed that exogenous ICL only significantly inhibited the proliferation, invasion and migration in primary tumor cells of LC patients with COPD compared to LC patients without COPD. Mechanism studies have shown that ICL could suppress the activation of NF-κB by blocking the hsa-miR19-3p/NKRF/NF-κB pathway as a microRNA sponge. Furthermore, In vivo experiments showed that exogenous ICL effectively inhibited the growth of patient-derived subcutaneous tumor xenografts (PDX) of LC patients with COPD and significantly prolonged the survival time of tumor-bearing mice. In a word, our study shows that the decrease of ICL is associated with an increased risk of LC in patients with COPD, ICL is not only expected to be a new therapeutic target for LC in COPD patients, but also has great potential to be used as a new marker for evaluating the occurrence, severity stratification and prognosis of LC in patients with COPD.

## Introduction

Chronic obstructive pulmonary disease (COPD) is the third leading cause of death in humans (about 11.7%) and is on the rise worldwide. Lung cancer (LC) is the most common tumor, ranking first in the cancer incidence and mortality in the world [1, 2]. Several clinical epidemiological studies have confirmed that the coexistence of COPD and LC is common, and these two diseases share many of the same risk factors. Once the lung cells of patients with COPD become cancerous, the tumors often obtain more rapid progress compared to that of LC patients without COPD [3]. LC patients with COPD often have a worse prognosis than LC patients without COPD [4]. Therefore, COPD is considered to be an independent risk factor for LC. One study observed the incidence rate and related risk factors of LC with the median follow-up period of 60 months in 2,507 COPD patients have shown that the incidence of LC was 16.7/1,000 patient-years, indicating that LC was more likely to occur in COPD patients than in others [5]. Another study of meta-analysis included 35 studies of 22,010 patients with COPD and 44,438 controls from 1980 to 2010, have shown that COPD was a high-risk factor for LC (OR = 2.76, 95% CI, 1.85–4.11) [6]. Together, these studies indicate that the thorough study of the pathogenesis of LC in a special group of patients with COPD and development of new targets is still an urgent requirement to reduce the high incidence of LC in patients with COPD, and is also the important means to improve the clinical treatment effect and prognosis of LC in COPD patients.

Recent studies have shown that the long non-coding RNA (lncRNA) can participate in the regulation of COPD in a variety of ways. LncRNA-SAL‑RNA1/2 can regulate the senescence of type II alveolar epithelial cells in patients with COPD through SIRT1/FOXO3a and SIRT1/P53 signaling pathways [7]. LncRNA-TUG1 was confirmed to be involved in the regulation of abnormal activation of pulmonary epithelial cells in patients with COPD [8]. Transcriptome sequencing analysis showed that there were 67 lncRNAs (33 up and 34 down) in peripheral blood mononuclear cells from patients with COPD [9]. In the meantime, accumulating evidence has revealed that lncRNAs participate in tumorigenesis and progression of LC and may represent compelling therapeutic targets in the treatment of LC. Knockdown of lncRNA-H19 decreased the colony formation ability and anchorage independent growth of LC cells [10]. Li et al. reported that lncRNA-CASC15 exhibits an oncogenic role in promoting non-small-cell lung cancer (NSCLC) tumorigenesis via regulating Epithelial-mesenchymal transition (EMT) [11]. MALAT1 is a lncRNA with more than 8000 base conserved among different species, it is expressed in a wide variety of tissues in the adult such as lung, pancreas and liver. Functional studies showed that down-regulation of MALAT-1 could inhibit the viability, proliferation, invasion, and cell cycle and promote apoptosis in A549 cells by inhibiting autophagy [12–14]. These studies make us more eager to know whether there is a certain lncRNA that mediates the relationship between LC and COPD. In addition, lncRNA is also involved in the regulation of maturation, differentiation and activation in innate immune cells.

Here, we report for the first time that lncRNA-ICL (Inhibitor associated with COPD and lung cancer), located on human chromosome 17 and significantly decreased in tumor tissues of LC patients with COPD compared to LC patients without COPD, may contribute to progression of LC in COPD patients. ICL is located on human chromosome 17, its function has not been reported in the past. Up to now, there is no report on the biological function of ICL. ICL is the important factor that we first obtained through high-throughput screening and confirmed to be beneficial to maintain the relationship between COPD and LC. In this study, we will focus on action pathway of ICL in promoting LC through in vitro and in vivo experiments, expecting to provide a theoretical support for it was used as a new therapy target and a new marker for prevention and treatment of LC in COPD patients.

## Materials and methods

### Ethical declarations

Informed consents were obtained from all the patients before surgery and the study was approved by the Ethics Committee of the Shanghai East Hospital’s Ethics Committee (NO.2020 − 182). Animal care and all experimental protocols involving mice were approved by Tongji University animal care and use committee (NO.2018-069) and carried out in accordance with the National Institutes of Health Guide for the Care and Use of Laboratory Animals.

### Tumour tissues

Twenty-four LC tissues were collected from LC patients with or without COPD who had not undergone any previous surgery or chemotherapy (12 cases in each group, n = 12) in the Department of Respiratory and Critical Care Medicine in Shanghai East Hospital from June 12, 2021 to November 11, 2021. The fresh tissues were collected and quickly divided into three parts within 2 h of surgery, one part is used to prepare primary LC cells and establishment of Patient-Derived Tumor Xenograft (PDX) model, the second is preserved with liquid nitrogen and the and third part is fixed with 4% paraformaldehyde. Clinicopathological characteristics of the patients and tumor classification according to the 2003 American Joint Committee on Cancer TNM classification is provided in Table 1. Subsequently, about 50 mg of frozen tissues were used to extract total RNA which were used for the measurement of ICL, hsa-miR19-3p, NF-κB p65 and NKRF mRNA levels using real-time quantitative PCR (RT-qPCR). Total protein extracted from about 100 mg tissues were used for detecting the proteins expression of NF-κB p65 and NKRF by using western blotting. The fixed tumor tissues were embedded in paraffin and used for detection of ICL and hsa-miR19-3p using fluorescence in situ hybridization (FISH) with the following probes, 5’-ACACGTTTAGATACGTT-3’ (5’/3’ Cy3 modification) for ICL and 5’-TCAGTTTTGCATAGATTTGCACA-3’ (5’/3’ DIG modification) for hsa-miR-19-3p according to a standard protocol [15].

**Table 1:** Informations of patients

No.	Gender	Age	Pathological	Lung cancer TNM stage
1	M	54	Squamous carcinoma	T_2_N_1_M_0_, II
2	M	60	Squamous carcinoma	T_3_N_2_M_0_, III
3	F	64	Adenocarcinoma	T_3_N_2_M_1_, IV
4	M	58	Adenocarcinoma	T_3_N_2_M_1_, IV
5	F	52	Adenocarcinoma	T_2_N_2_M_0_, III
6	F	68	Squamous carcinoma	T_4_N_2_M_1_, IV
7	M	70	Squamous carcinoma	T_1_N_1_M_0_, II
8	M	72	Squamous carcinoma	T_3_N_2_M_1_, IV
9	F	70	Adenocarcinoma	T_3_N_3_M_0_, III
10	F	69	Adenocarcinoma	T_2_N_1_M_0_, II
11	F	53	Squamous carcinoma	T_2_N_3_M_0_, III
12	M	73	Adenocarcinoma	T_1_N_1_M_0_, II
13	M	70	Squamous carcinoma with COPD	T_3_N_2_M_0_, III
14	F	69	Squamous carcinoma with COPD	T_3_N_2_M_1_, IV
15	F	59	Adenocarcinoma with COPD	T_3_N_3_M_1_, IV
16	M	63	Squamous carcinoma with COPD	T_3_N_3_M_0_, III
17	F	66	Squamous carcinoma with COPD	T_1_N_1_M_0_, II
18	F	72	Adenocarcinoma with COPD	T_4_N_2_M_1_, IV
19	F	58	Adenocarcinoma with COPD	T_4_N_2_M_1_, IV
20	M	60	Squamous carcinoma with COPD	T_3_N_3_M_0_, III
21	M	63	Squamous carcinoma with COPD	T_3_N_2_M_0_, III
22	M	65	Squamous carcinoma with COPD	T_4_N_3_M_1_, IV
23	M	72	Squamous carcinoma with COPD	T_2_N_1_M_0_, II
24	M	72	Adenocarcinoma with COPD	T_3_N_3_M_1_, IV

The diagnosis of COPD was made by experienced physicians based on Global Initiative for Chronic Obstructive Lung Disease criterion. The international tumor-node-metastasis (TNM) staging system is the “international language” in cancer diagnosis and treatment. Patients with lung cancer were classified according to the eighth edition of the TNM classification

### Cell culture

Primary LC cells were prepared from LC tissues according to erreño’s protocol and were named LCs (from LC patients without COPD) or C-LCs (from LC patients with COPD) respectively [16]. Briefly speaking, the fresh LC tissues were quickly cut it into 3 × 3 mm pieces by using sterile surgical scissors. Tissue samples were rinsed twice with 1 mL sterile phosphate-buffered saline. Then, the tissue was immersed in RPMI1640 culture medium (Thermo Fisher Scientific, CA, USA) supplemented with 5% fetal bovine serum (FBS, Thermo Fisher Scientific) and 1% streptomycin/penicillin (Thermo Fisher Scientific). Under a stereoscope, samples were mechanically fractionated by cutting with scissors or scalpel, and fragments were cultured into 35-mm dish. The cells were resuspended in 300 µL RPMI1640 culture medium supplemented with 10% FBS. Once aconfluent monolayer was obtained, cells were harvested with 0.25% trypsin (EDTA+) and seeded into 60-, and 100-mm culture dishes for serial passages. 293T cells used as tool cells for luciferase analysis and lentivirus production were purchased from American Type Culture Collection (ATCC, VA, USA) and maintained in Dulbecco’s modified Eagle’s medium (DMEM, Thermo Fisher Scientific) containing 10% FBS. All the adherent cells were cultured in an atmosphere with saturated humidity at 37 °C containing 5% CO2. At 80% confluence, the cells were subcultured at a ratio of 1:3 with 0.25% trypsin.

### Preparation of recombinant lentivirus

An siRNA (5’-GAACCTTTCTAATCCAGAA-3’) targeting to the coding sequence (CDS) of human NKRF(NM_001173487.1) was selected for construction of pshRNA-NKRF which carry an H1 promoter used to drive siRNA expression, and the scrambled sequence of siRNA (5’- CTCAGAATACTGACACTTA-3’) was used as negative control (NC) to construct pshRNA-NC. The ICL amplified using human complementary DNA (cDNA) as a template with the PCR primers 5’-TTGGGAGCTGGCTGCTGCGCCCAG-3’(forward) and 5’- CGCGGCAGCTGTTGCCCCGGGATTATT-3’ (reverse), was used for the construction of expression vector pcDNA-ICL which carry an CMV promoter used to drive ICL expression. All the expression vectors have Green Fluorescent Protein (GFP) gene used as the marker for evaluation of transfection efficiency.

A total of 1 × 10^6^ logarithmic 293T cells purchased from American Type Culture Collection (ATCC, VA, USA) were inoculated plated into 10-cm dishes in 10 mL DMEM medium supplemented with 10% FBS and cultured overnight under normal conditions. Recombinant vectors the lentivirus packaging plasmids were co-transfected into 293T by using Lipofectamine 2000 (Thermo Fisher Scientific). The culture medium was completely replaced with DMEM containing 1% FBS prior to transfection. After 48 h of transfection, the supernatant was harvested and cleared by centrifugation at 5,000 × g for 10 min at 4 °C and then passed through a 0.45 μm polyvinylidene difluoride membrane (Millipore, MI, USA). Viral titers were determined using the gradient dilution method. The recombinant lentiviruses were named Lv-ICL, Lv-shRNA-NKRF and Lv-NC, respectively.

### Dual-luciferase reporter assay

#### Verification the binding sites of has-miR19-3p in NKRF − 3’ untranslated region (UTR)

We used online software “Targetscan7.1” (http://www.targetscan.org/) to predict the binding site of hsa-miR19-3p in the 3’-UTR of human NKRF. Then the 3’-UTR of the NKRF (169 bp) containing the hsa-miR19-3p target sites were amplificated by using human cDNA as the template with primers 5’ -TTCCCATGGCCATTTCTGTGGAGG-3’ (forward) and 5’-CCTGAGTGGGGTGGGAGCTT-3’ (reverse). The PCR product was cloned into pGL3-promoter vector (Promega Corporation, WI, USA) downstream of the firefly luciferase gene to generate pGL3-wt (wild-type)-NKRF carrying a wild target site 5’- TTTGAAC-3’. Then the target site of hsa-miR19-3p in pGL3-wt-NKRF was mutated to construct pGL3-mt-NKRF carrying a mutation target site 5’-TTGATAC-3’ by using a site-directed mutagenesis kit (Takara Bio Inc. Dalian, China). The hsa-miR19-3p mimics (5’-UGUGCAAAUCUAUGCAAAACUGAtt-3’), inhibitor (5’-UCAGUUUUGCAUAGAUUUGCACAtt-3’), and NC (5’-AGAUUGCCGCUAAUAAAAUCUGAtt-3’) were chemically synthesized by Shanghai GenePharma Co., Ltd (Shanghai, China). 293T cells inoculated in 24-well plate and cultured overnight were co-transfected with the hsa-miR19-3p mimics, inhibitor, NC, and pGL-wt-NKRF or pGL3-mt-NKRF using Lipofectamine 2000. Forty-eight hours after transfection, cells were lysed and used for luciferase assays using the Dual Luciferase Reporter Assay System (Promega). Finally, we can speculate whether hsa-miR19-3p can bind to 3’UTR of NKRF by binding to the predicting sites according to the change of luciferase activity in cells, Meanwhile, the inhibitory effect of ICL on the binding of hsa-miR19-3p to NKRF 3’UTR was evaluated by observing the effect of ICL overexpression on luciferase activity in 293T cells co-transfected with hsa-miR19-3p mimics and pGL3-wt-NKRF. In addition, we will also co-transfect hsa-miR19-3p mimics, pGL3-wt-NKRF and pcDNA-ICL into 293T cells, and evaluate whether ICL affects the binding of hsa-miR19-3p to 3’UTR of NKRF according to the effect of pcDNA-ICL on the intracellular luciferase activity.

#### Validation of the transcription factor binding site (TFBS) of NF-κB in the promoter of hsa-miR19-3p

We firstly checked the location of the precursor of hsa-miR19-3p (pri-miR19) in the human genome and selected a 2.0 kb DNA sequence upstream of the transcription start site as the promoter region according to conventional practice. Then we used software Promoter 2.0 (DTU Health Tech, Lyngby, Denmark) to predicted promoter of hsa-miR19-3p which was amplified using human genomic DNA as the template with the primers 5’-GGCTCGGCGGGAGCGGCGTCCCCG-3’(forward) and 5’-GG GGCAGGAACACCCGAGACTGCAA-3’ (reverse) .We then cloned the promoter upstream of GFP gene to construct a fluorescent expression vector pcDNA-pro(miR19-3p)-GFP which was transfected in to 293T cells. Forty-eight hours after transfection of pcDNA-pro(miR19-3p)-GFP, the expression of GFP was observated in cells using an inverted fluorescence microscope (IX71, Olympus Corporation, Japan) and was used to evaluate whether the promoter guide downstream gene transcription. An expression vector pcDNA-pro (-/-)-GFP with the predicted promoter removed was used as the negative control. After confirming the hsa-miR19-3p promoter, the TFBS of NF-κB in the hsa-miR19-3p promoter was predicted using applied bioinformatics software “JASPAR” (http://jaspar.genereg.net). Then we cloned the promoter into the pGL3-Enhancer (Promega) upstream of the luciferase gene to construct pGL3-TFBS (wt)-miR19-3p which carrying the wild-type TFBS 5’-CGGAGCCCCC-3’. Then, the TFBS in the pGL3-TFBS (wt)-miR19-3p vector was mutated to construct pGL3-TFBS (mt)-miR19 which carrying a mutated TFBS 5’-ACGGCCGCCC-3’. The luciferase pGL3-TFBS(wt)-miR19 or pGL3-TFBS(mt)-miR19 were transfected into 293T cells followed by induction with 5 ng/ml Tumor Necrosis Factor Alpha (TNF-α, Abcam, Cambridge,UK) for 48 h to activate intracellular NF-κB. Forty eight hours after transfection and induction, the cells will be used for luciferase activity detection which were used to confirm whether NF-κB can regulate the transcription of pri-miR19 via the predicted TFBS.

### Chromatin immunoprecipitation (ChIP)

To further confirm the binding of NF-κB to promoter of hsa-miR19-3p, the ChIP assay was carried out using the EZ ChIP Kit (Millipore, MI, USA) according to the manufacturer’s instructions. The C-LCs cells cultured in 10-cm dishes were crosslinked with 1% formaldehyde in PBS and plates were incubated on a rotator for 10 min at 25°C. Subsequently, formaldehyde was removed and crosslinking quenched by incubation with 125 mM glycine in PBS for 5 min at 25°C. Following solution removal, plates were chilled on ice and the cells lysed by adding 2 mL of cold lysis buffer with a protease inhibitor (Roche, USA). The chromatin was fragmented to 200–500 bp with a Misonix S3000 Sonicator (Farmingdale, USA) at 4°C. After centrifugation at 10,000 × g for 5 min at 4°C, chromatin supernatants were diluted with cold IP dilution buffer. The human NF-κB-p65 antibody (2 µg, Abcam) was added to chromatin and the mixture was incubated at 4°C overnight. Dynabeads were added to the chromatin/antibody mixture and incubated for additional 4 h at 4°C. Beads were washed with the wash buffer and samples were eluted with 250 µl elution buffer. The eluted samples were treated with 0.2 M NaCl and 1 mg/mL Protease K at 65°C overnight. Chip samples were purified with phenol/chloroform and precipitated with cold ethanol and glycogen. Then the reverse transcription-polymerase chain reaction (RT-PCR) was performed to amplify a 52 bp PCR product carrying the predicted TFBS of NF-κB on the TP650 system (Takata) using a Takara Ex Taq (RR001Q, Takara) according to the manufacturer’s instructions. Five microliters of input or eluents was used as the templates. PCR reaction parameters were as follows: 95°C for 10 s, 60°C for 20 s, 72°C for 20 s, 45 cycles. The primer sequences used were as follows: 5’-GCCTCGGGCCGCGTGCGACG-3’(forward) and 5’-GGCCCAGAGGGGCGGGGGCTCCGCG-3’ (reverse). After PCR reaction, each PCR product was taken at 5 µL for 2% agarose gel electrophoresis.

### Cell proliferation, invasion and migration assays

After 72 h of infection by Lv-NC or Lv-ICL, the C-LCs were used for cell viability, invasion and migration assay. The C-LCs of 2nd generation in logarithmic growth period were re-inoculated to 96-well plate or 6-well plate with 5.0 × 104/well or 2.0 × 10^5^/well using RPMI1640 medium containing 10% FBS, and then were cultured overnight at 37 ℃ and 5% CO2. After that, the lentivirus was added to the medium with the multiplicity of infection (MOI) of 10. Then the cells in 96-well plate were continued to be cultured under normal conditions for 72 h and used for proliferative activity assay by CCK-8 method, and cells in 6-well plate cultured under normal conditions for 48 h were used for invasion and migration analysis. The cell viability assay was assessed using a Cell Counting Kit-8 kit (CCK-8, CK04, Dojindo, Japan) according to the instructions. Briefly, 10 µl of CCK-8 solution was added to each well of 96 well plate, and then the cells were cultured under normal conditions for an additional 2 h. Then the absorbance at 450 nm was measured. Cellular invasion was assessed using QCMTM 24-well Fluorimetric Cell Invasion Assay kit (ECM554, Chemicon International, WI, USA) according to the manufacturer’s instructions. Briefly, after 72 h of infection. C-LCs were seeded at a density of 4.0 × 10^4^ cells per well in 500 µL of serum-free RPMI1640 in 0.8 μm trans-well chambers. Next, 750 µL of complete medium with 10%FBS was added in to 24-well plates and incubated at 37 °C with 5% CO2. The following day, the cells on the top surface of the insert were scraped off, and the cells on the bottom surface were fixed with ice-cold methanol followed by hoechst33342 (ab228551, Abcam) staining. The number of cells was counted using light microscopy, and the data are presented graphically. The migration was assessed using the wound scratch assay in LCs and C-LCs. Cells were seeded into six-well plates (1.0 × 10^5^ /well). When the cells grew to 90% confluence, the cell monolayer was scrape-wounded with a sterile 200 µL pipette tip and floating cells were washed away using dPBS. The remaining cells were incubated for 24 h. Scratched cells were visualized and images were captured under the inverse microscope at 0 and 24 h after scratching referring to previous published report [11].

### Effect of exogenous ICL on the proteins involved in LC development

The C-LCs of 2nd generation in logarithmic growth period were seeded into 6-well plates in 1 mL of RPMI1640 medium with 10%FBS at a density of 1.0 × 10^5^ cells/well and cultured under normal conditions. One day later, lentiviruses (Lv-NC, Lv-ICL and Lv-shRNA-NKRF) were added to the medium at a MOI of 10 and cultured under normal conditions. The infection efficiency was assessed by observing the fluorescence of GFP 72 h after infection. Then the total RNA was isolated from the cells and subjected to RT-qPCR to analyze the levels of ICL and hsa-miR19-3p. The cells were also used to isolate proteins which were used for the measurement of phosphorylation of NF-κB-p65 and expressions of proteins NKRF, BCL-2, CyclinD1 and VEGF by western blotting.

### Animal xenografts

Tumor tissues obtained from six LC patients with COPD were temporarily stored in RPMI1640 medium containing 10% FBS and 1% penicillin and streptomycin pre-cooled at 4 ℃. The tumor tissues were cut into 2 × 2 × 2-mm pieces before they were subcutaneously engrafted into NSG mice (SHANGHAI MDDEL ORGANISMS, Shanghai, China) to establish PDX mode. The tissues were transplanted subcutaneously in the right flank of 6-week-old male mice. When xenografted tumors grew to approximately 5 × 5 × 5-mm3, we followed the aforementioned protocols to harvest the tumors from 6 mice (n = 6) and transplanted them subcutaneously in the right flank of 6-week-old male mice to establish next-generation PDX mode, one tumor was inoculated into 5 mice. After about two weeks, the tumor in the second generation of tumor bearing mice grew to about 5 mm in diameter. Follow up experiments were carried out from 3 mice with uniform tumor size corresponding to each patient which were divided into three groups. The number of samples from each group was 6, corresponding to six patients respectively (n = 6). Model group (were given tail vein injection of equal volume PBS with intervention groups), Lv-NC group (were given tail vein injection of Lv-NC of 10^7^ifu/mouse), and Lv-ICL group (tail vein injection of Lv-ICL of 10^7^ifu/mouse), the administration lasted for 6 weeks once a week. Growth of the established tumor xenografts was monitored at least twice weekly through measurement of the length (a) and width (b) of the tumor. The tumor volume was calculated as (a×b×b)/2. After 5 weeks of administration, the tumors were stripped and divided into two parts. One was immediately snap frozen and stored in liquid nitrogen until RNA and protein extraction which were used for detecting expression of ICL and miR19-3p, and phosphorylation of NF-κB-p65 by RT-qPCR and western blotting. The second part was fixed with paraformaldehyde for immunofluorescence staining of CD34 and immunohistochemistry staining of Ki67. For immunofluorescence analysis, the fixed tumor tissues were processed and embedded in paraffin using standard methods. Then they cut into 4-µm thick slices, and deparaffinized. For antigen retrieval, slices were immersed in EDTA-tris solution (pH = 9.0) for 30 min at 96 °C. Tissue sections were then blocked with 10% normal goat serum in 0.01 M phosphate-buffered saline at room temperature for 1 h and incubated with anti-CD34 (ab81289,1:200, Abcam) overnight at 4 °C, followed by incubation with Goat Anti-Rabbit IgG (Alexa Fluor488) (ab150077,1:3000, Abcam) for 2 h at 37 °C. After counterstaining with 4,6-diamidino-2-phenylindole (DAPI) for 8 min at 37 °C, the immunostaining was visualized by fluorescence microscopy at 200× magnification. For immunohistochemistry staining for Ki67, 4 μm thick sections of the tissues were cut, deparaffinized in xylene and rehydrated with graded alcohol. The sections were washed with phosphate buffered saline (PBS) and then boiled in citrate buffer (pH = 6.0) for 15 min. The sections were incubated with 3% H_2_O_2_ for 5 min to block the endogenous peroxidase activity and then incubated in goat serum to decrease nonspecific staining. The sections were incubated with anti-Ki67 antibody (ab92742,1:300, Abcam) overnight at 4 °C, incubated with secondary antibody Goat Anti-Rabbit IgG (HRP) (ab6721, 1:2000, Abcam) at 37 °C for 20 min and then treated with DAB (ab64238, Abcam) for 10 min. The sections were counterstained with hematoxylin-eosin for 3 min and then dehydrated in ethanol. To analyze the Ki67 positive rate, the labeling index was performed. Three random images from every cell slide were photographed in triplicate. The labeling index was calculated by dividing the number of Ki67 positive cell nuclei by the total number of cells and multiplying by 100.

### Survival analysis of subcutaneous tumor-bearing mice

Thirty-six subcutaneous tumor-bearing mice (6 mice corresponds to one case) were generated according to the inoculation method mentioned above, the animals were randomly divided into 3 groups (12 mice/group), and the survival rate of the animals was monitored for 12 weeks. The gene intervention method was identical to the method used for the tumor formation experiment and continued until the 12th week. Once the subcutaneous tumor reached 20 mm in length, it was recorded as a death event, and the data were used to analyze the survival period of the tumor-bearing animals [16].

### RT-qPCR

Total RNA was isolated form cells and tissues by using TRIzol reagent (15596026, Thermo Fisher Scientific) and reverse-transcribed into cDNA using M-MLV reverse transcriptase (2641A, Takara). Subsequently, PCR was performed using TB Green Premix Ex Taq (RR820B, Takara) in a total volume of 20 µL using amplification condition of at 95°C for 5 min, followed by 40 cycles at 95°C for 10 seconds and 55°C for 30 seconds on ABI7500Fast (Thermo Fisher Scientific). Results were analyzed by 2^−∆CT^ analysis. For detecting of ICL and NKRF mRNA levels, β-actin was used as a reference gene. The PCR primers used were: ICL, 5’-CCTCTCTTTGAACTTGAAATGC-3’(forward) and 5’-TCCTGGGTCTGAGCATTAGAACCA-3’ (reverse), 5’-AAGACCAGCCTGTAACAGCCAA-3’ (forward) and 5’-TTGCCTGCTGTGATGTGG-3’(reverse) and β-actin, 5’-CCTGTACGCCAACACAGTGC-3’ (forward) and 5’-ATACTCCTGCTTGCTGATCC-3’ (reverse). For normalizing the level of hsa-miR19-3p, U6 snRNA was used as reference. The specific primers used for reverse transcription were random9-mer for the U6 snRNA and 5’-GTCGTATCCAGTGCGTGTCGTGGAGTCGGCAATTGCACTGGATACGATCAGT-3’ for hsa-miR19-3p.

### Western blotting

Total protein was extracted from cells using the M-PER mammalian protein extraction reagent and from tissues using the T-PER tissue protein extraction reagent (78501,78510, Thermo Fisher Scientific). Equal amounts of total proteins (15 µg) were separated by sodium dodecyl sulfate polyacrylamide gel electrophoresis (SDS,11% gel) and transferred to nitrocellulose membranes. The blots were probed with primary antibodies against human p-NF-κB-P65 (ab76032,1:300), NF-κB-P65 (ab16502,1:600), NKRF (ab168829, 1:500), Bcl-2 (ab241548, 1:500) and β-actin (ab5694,1:1000) (Abcam), then followed by probing with Goat Anti-Rabbit IgG H&L (HRP) (1:3000, ab6721, Abcam) secondary antibody. After the membranes were washed, the protein blots were detected with the application of an enhanced chemiluminescent (ECL) Chemiluminescence Detection Kit (32,134, Thermo Scientific) and subjected to analysis by Image Lab Software (Bio-Rad, Hercules, CA, US). β-actin was used as an endogenous reference for normalization. The evaluation of protein phosphorylation was based on its own total protein (phosphorylation + non-phosphorylation).

### Statistical analysis

Data analysis was performed using SPSS 24.0 statistical software (SPSS Inc., Chicago, IL, United States). The data are shown as the means ± SD (standard deviation, SD) of three independent experiments, and *P* < 0.05 was be used as the criterion for statistical significance. In pairwise comparisons between groups, t-tests were used for two independent samples with homogeneity of variance. Comparisons between groups were performed using a two-tailed Student’s *t*-test or one-way ANOVA with a post-hoc Tukey test.

## Results

### The levels of ICL were significantly decreased in LC tissues of LC patients with COPD compared to LC patients without COPD

**Fig. 1:**
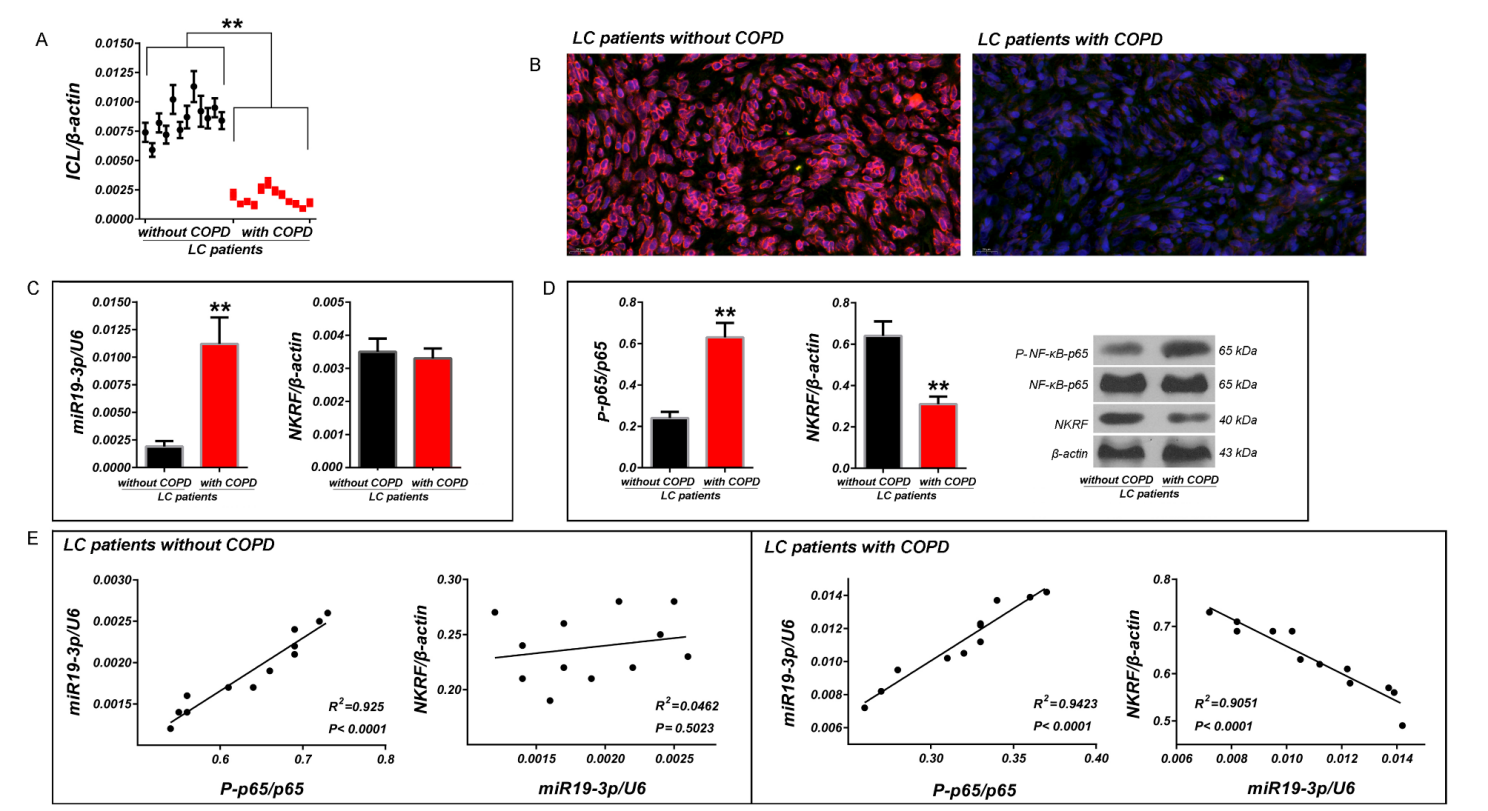
Detection of expression of ICL, hsa-miR19-3p, NKRF and phosphorylation of NF-κB-p65 in LC tissues of LC patients with and without COPD and their correlation analysis. (**A**) The relative expression of ICL were detected in LC tissues by RT-qPCR. (**B**) Detection of ICL in LC tissues by FISH. (**C**) The relative levels of hsa-miR19-3p and NKRF mRNA were detected in LC tissues by RT-qPCR. (**D**) Phosphorylation of NF- κB-p65 and expression of NKRF protein in LC tissues were detected by western blotting. (**E**) Spearman correlation analysis between phosphorylation NF-κB-p65 and hsa-miR19-3p, hsa-miR19-3p and NKRF protein in LC tissues. RT-qPCR was performed using U6 and β-actin as references, respectively, and the 2^−ΔCt^ method was used for analysis of inter-group difference. For western blotting, β-actin was used as the reference. ^**^p < 0.01 and ^*^p < 0.05. All data are expressed as the mean ± SD (sample size, n = 12)

In order to re-check the data in the previous screen, the relative quantitative analysis of ICL we carried out in LC tissues of LC patients with and without COPD by using RT-qPCR. The results showed that ICL were significantly decreased in LC tissues of LC patients with COPD than that of LC patients without COPD (*p* < 0.01 vs. LC patients without COPD, Fig. 1A). The FISH results showed that the detection signal of ICL (Cy3-red) was significantly weaker in LC tissues of LC patients with COPD than that of LC patients without COPD (Fig. 1B).

### Hsa-miR19-3p maintained a positive correlation with phosphorylation of NF-κB p65 in LC tissues of patients regardless of COPD, but a negative correlation between it and NKRF protein was only maintained in LC tissues of patients with COPD

Our previous co-expression analysis had shown that ICL had a significant negative correlation with the downstream regulatory genes of nuclear transcription factor NF-κB such as Bcl-2, CylinD1, and VEGF. Further detection showed that the phosphorylation of NF-κB-p65, rather than the expression of protein, was negative correlated with ICL in LC tissues. Therefore, we speculate that ICL may participate in the regulation of NF-κB activity through the interaction with NF-κB inhibitors. Subsequently, we detected the protein expression of I-κBα and NKRF, the most two common inhibitors of NF-κB. The correlation analysis showed that the expression of NKRF in LC tissue of patients with COPD was significantly lower than that of LC patients without COPD, and the difference only existed at the post-transcriptional level (mRNA content remained unchanged). This made us believe that the abnormal expression of NKRF might be attributed to the posttranscriptional regulation. As a typical post-transcriptional regulation mechanism, we quantitatively detected miRNAs upstream of NKRF, and hsa-miR-19-3p showed a good negative correlation with NKRF protein in LC tissue of patients without COPD, which is consistent with the theory that miRNA negatively regulates gene expression at post-transcriptional level. Interestingly, it seemed that hsa-miR-19-3p lost its negative regulation on NRKF protein in LC tissue of patients with COPD. Combined with the latest progress in miRNA function research, we believed that NF-κB p65, miR-19 and NRKF should have an interwoven regulatory network in the progression of LC in patients with COPD. RT-qPCR data showed that hsa-miR19-3p were significantly increased in LC tissues of patients with COPD than that of LC patients without COPD (*p* < 0.01 vs. LC patients without COPD), and there was no significant change in the mRNA level of NKRF between the two groups (*p*>0.05, Fig. 1C). Western blotting showed that expression of NKRF was decreased significantly in LC tissues of patients with COPD than that of LC patients without COPD (*p* < 0.01 vs. LC patients without COPD), and phosphorylation of NF-κB p65 were significantly enhanced in LC tissues of patients with COPD than that of LC patients without COPD (*p* < 0.01 vs. LC patients without COPD. Figure 1D). Comprehensive analysis revealed that hsa-miR19-3p was positively correlated with phosphorylation of NF-κB p65, and negatively correlated with NKRF protein in LC tissues of patients with COPD. However, in LC tissues of patients without COPD, hsa-miR19-3p was only positively correlated with phosphorylation of NF-κB p65 (Fig. 1E).

### Hsa-miR19-3p can inhibit protein expression of NKRF by interacting with the 3’-UTR of protein coding mRNA

**Fig. 2:**
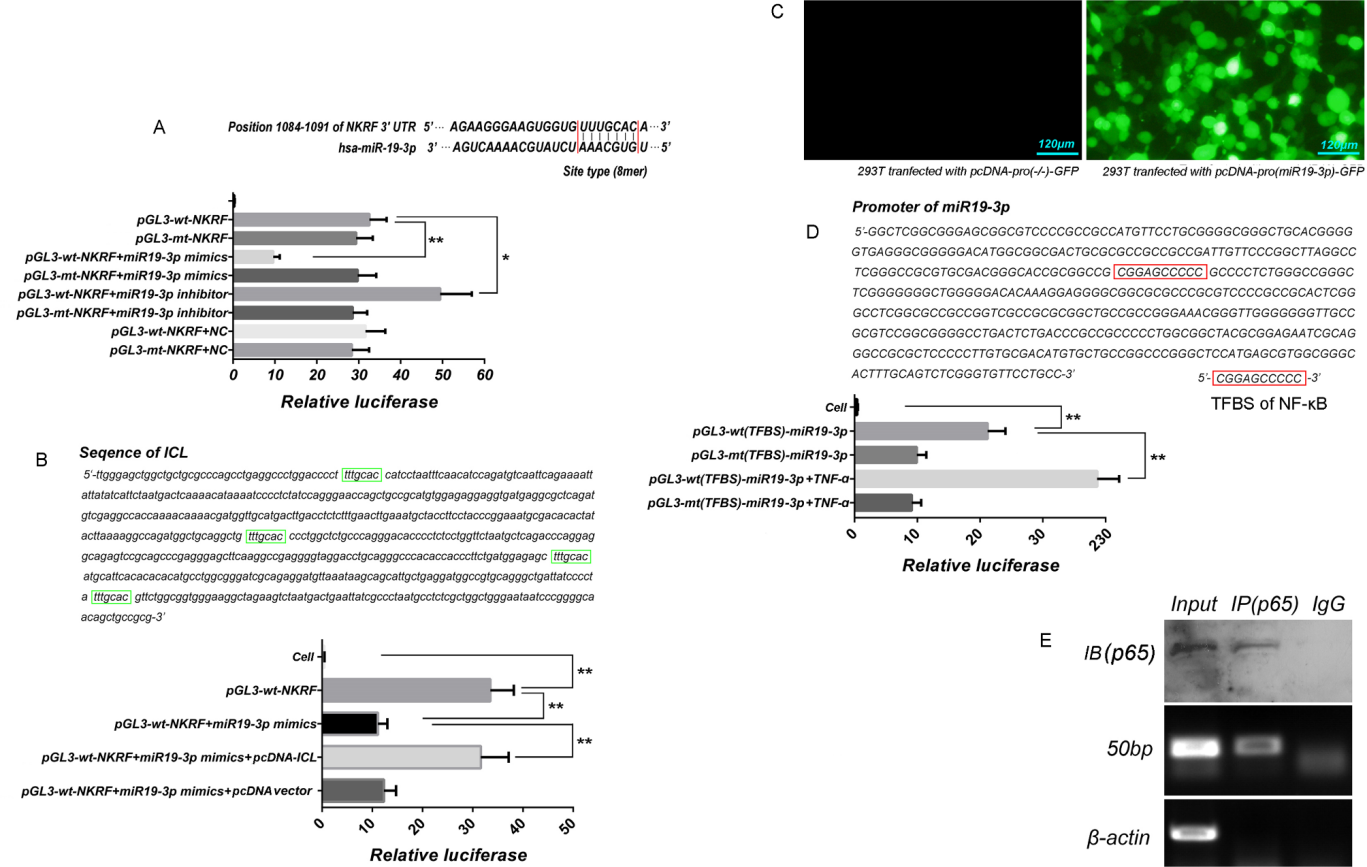
Luciferase and CHIP assay. (**A**) The luciferase experiment was used to evaluate the binding sites of hsa-miR19-3p to 3’-UTR of NKRF. Upper, prediction of binding sites of hsa-miR19-3p to 3’-UTR of NKRF. Down, analysis of differences between groups. The histogram shows the relative activity of luciferase. *, *p* < 0.05, and **, *p* < 0.01. (**B**) The luciferase experiment was used to evaluate whether ICL can weaken the binds of hsa-miR-19-3p to NKRF 3’-UTR. Upper, prediction of binding sites of hsa-miR19-3p to ICL. Down, analysis of differences between groups. (**C**) Evaluation of the activity of hsa-miR19-3p promoter. 48 h after transfection, we can evaluate the promoter activity by observing the expression of GFP in 293T cells. (**D**)The luciferase experiment was used to evaluate whether there is a TFBS of NF-κB in the hsa-miR-19-3p promoter. TNF-α (5ng/mL) was used to activate NF-κB in 293T cells. If NF-κB could bind to miR-19-3p promoter, the addition of TNF-α would inevitably up-regulate the luciferase activity in 293T cells 48 h after transfection. (**E**) CHIP-PCR was used to verify the TFBS of NF-κB locus on the promoters of hsa-miR19-3p. RT-PCR was used to detect whether PCR amplification with different eluents as templates had the single product. Five microliters PCR products was taking for 2% agarose electrophoresis. β-actin was used as reference. The tests were carried out on three biological triplicates, and data are expressed as the mean ± SD. ** *p* < 0.01, * *p* < 0.05

Bioinformatics analysis identified a seven-base binding sites 5’-UUUGCAC-’3 of hsa-miR19-3p seeds region in the 3’-UTR of NKRF mRNA (Fig. 2A, upper panel). The luciferase data showed that the strong luciferase activity was detected in 293T cells infected with either pGL3-wt-NKRF or pGL3-mt-NKRF after 48 h of transfection (*p* < 0.01, vs. 293T cells untransfected). The hsa-miR19-3p mimics significantly inhibited luciferase activity in 293T cells transfected with pGL3-wt-NKRF from 32.54 ± 4.11 to 9.62 ± 1.52; *p* < 0.01, vs. 293T cells transfected with pGL3-wt-NKRF). However, the hsa-miR19-3p inhibitor significantly increased luciferase activity in these cells from 32.54 ± 4.11 to 49.43 ± 7.57; *p* < 0.05, vs. 293T cells transfected with pGL3-wt-NKRF). In contrast, neither the hsa-miR19-3p mimics nor inhibitor had any effect on luciferase activity in 293T cells transfected with pGL3-mt-NKRF (*p* > 0.05, *vs*. 293T cells transfected with pGL3-mt-NKRF). Transfection of hsa-miR19-3p-NC had no effect on the luciferase activity in 293T cells transfected with either of the two vectors (*p* > 0.05, vs. 293T cells transfected with pGL3-wt-NKRF or pGL3-mt-NKRF) (Fig. 2A, lower panel).

### ICL interferes with binding of hsa-miR19-3p to NKRF 3’-UTR

Bioinformatics analysis indicated the existence of four binding sites of hsa-miR19-3p in ICL (Fig. 2B, upper panel). Luciferase assay showed that hsa-miR19-3p mimics lost their inhibitory effect on luciferase activity in 293T cells transfected with pGL3-wt-NKRF following transfection of pcDNA-ICL (Fig. 2B, lower panel). This indicates that ICL can indeed suppress the binding of hsa-miR19-3p to the 3’UTR of NKRF mRNA by acting as the miRNA sponge.

### NF-κB can positively regulate the transcription of hsa-miR19-3p by binding to its promoter

Forty-eight hours after transfection, strong GFP expression was observed in most 293T cells transfected with pcDNA-pro(miR19-3p)-GFP using fluorescence microscopy, indicating the promoter of hsa-miR19-3p have the ability to activate the transcription of its downstream gene (Fig. 2C). We then obtained a 438 bp promoter of hsa-miR19-3p and a conserved NF-κB binding sequence, 5’-CGGAGCCCCC-3’ in the promoter by bioinformatics analysis (Fig. 2D, upper panel). Luciferase assays showed that induction of TNF-ɑ (5ng/ml) for 48 h significantly increased the luciferase activity in 293T cells transfected with pGL3-wt (TFBS)-hsa-miR19-3p (*p* < 0.01, vs. 293T cells transfected with pGL3-wt (TFBS)-hsa-miR19-3p), but had no effect on the luciferase activity in 293T cells transfected by pGL3-mt (TFBS)-hsa-miR19-3p (*p*>0.05, vs. 293T cells transfected with pGL3-mt (TFBS)-hsa-miR19-3p), indicating that NF-κB activates transcription of hsa-miR19-3p by binding to its promoter (Fig. 2D, lower panel). ChIP assay further validated the TFBS of NF-κB on hsa-miR19-3p promoter. The results of agarose gel electrophoresis showed that PCR amplification using input and eluent of NF-κB protein as templates could obtain fragments, indicating that NF-κB can bind to hsa-miR19-3p promoter through predicted TFBS (Fig. 2E).

### Exogenous ICL showed a stronger ability to inhibit proliferation, invasion and migration in LC cells of LC patients with COPD than that of LC patients without COPD

**Fig. 3:**
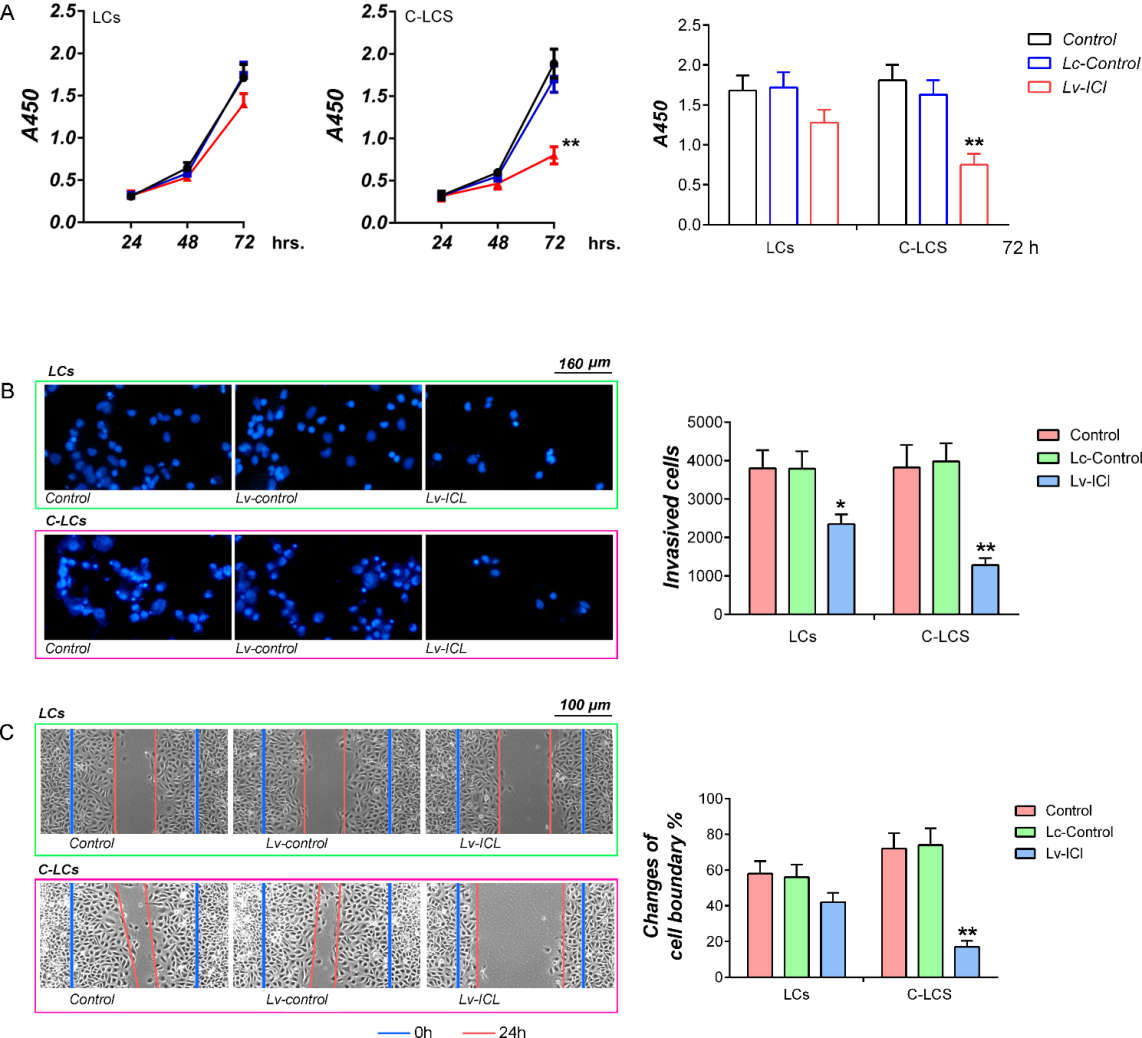
Analysis of the effects of ICL on proliferation, invasion and migration in LCs and C-LCs. (**A**) CCK-8 assay. The y-coordinate represents the absorbance of samples at 450 nm. (**B**) Cell invasion assay. Cell invasion assay was performed by trans-well method. The cells passing through the membrane were fixed with ice-cold methanol followed by hoechst33342 staining (Left). The number of cells was counted using light microscopy, and the data are presented graphically (Right). (**C**) Wound healing assay on the migration of LCs and C-LCs infected with Lv-control or Lv-ICL. The calculation formula of wound healing percentage (relative width) was as follows: (the width of wound at 0 h- the width of wound at 24 h)/the width of wound at 0 h. ** *p* < 0.01, * *p* < 0.05. The tests were carried out on three biological triplicates, and data are expressed as the mean ± SD.

In order to prove that ICL can promote the progression of LC in patients with COPD, we conducted a comparative study on the function of ICL in tumor cells between LC patients with and without COPD. Cell proliferation assay data showed that overexpression of ICL could effectively suppress the proliferation of LCs and C-LCs (*p* < 0.05 vs. LCs group. *p* < 0.01 vs. C-LCs group, 72 h.). However, the degree of inhibition of ICL on cell activity is significantly higher in C-LCs than in LCs (Fig. 3A). The data of invasion and migration assays showed that the change trend of cell invasion and migration in cells of each group was completely consistent with the change of proliferation activity (Fig. 3B, C).

### ICL can inhibit NF-κB activity which can be blocked by NKRF silencing

**Fig. 4:**
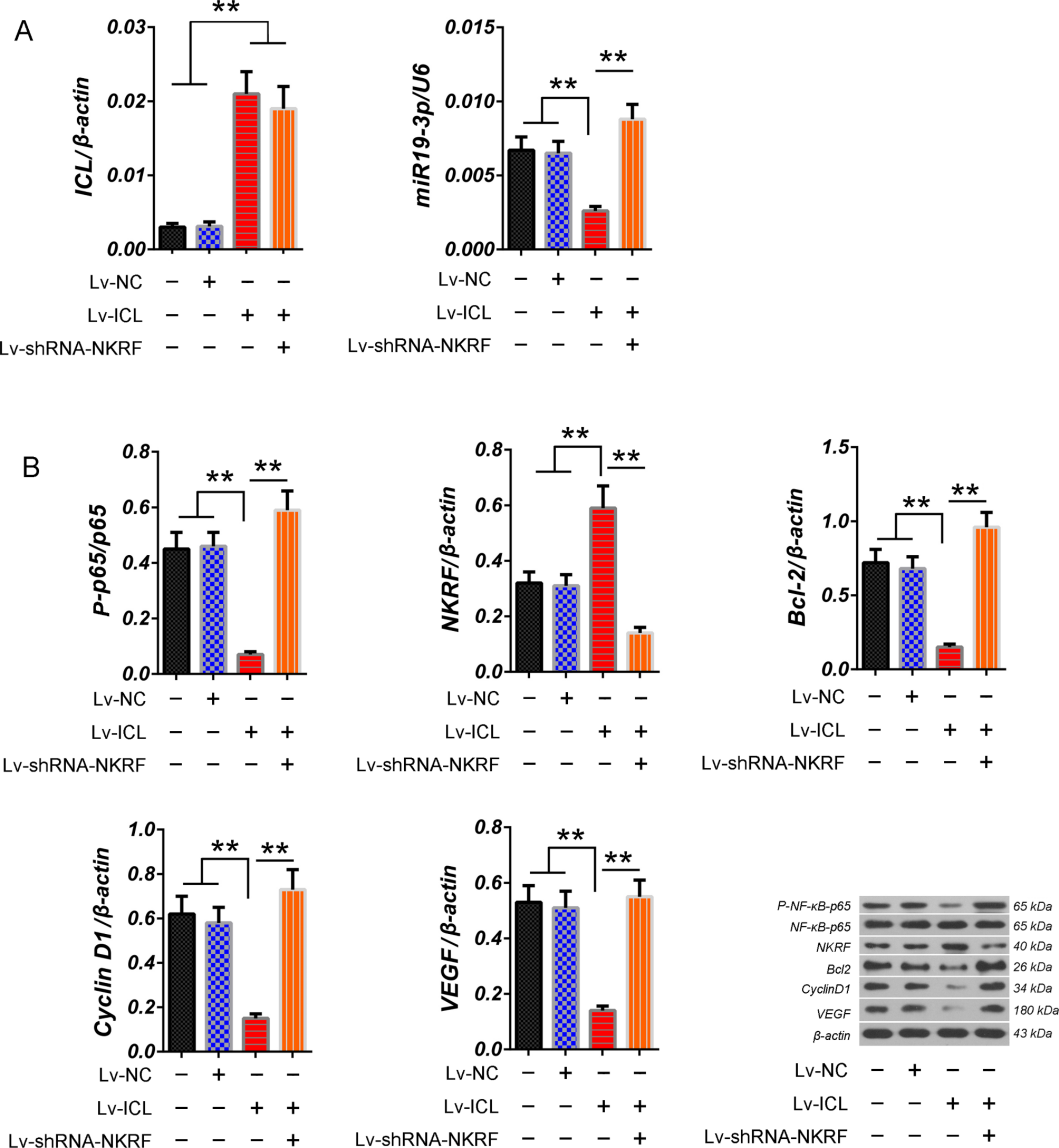
Analysis of the influence of overexpression of ICL on the expression or phosphorylation of mumbers of hsa-miR19-3p/NKRF/NF-κB pathway and the expression of tumor-related proteins regulated by NF-κB. (**A**) The relative levels of ICL and has-miR19-3p in C-LCs of each group were detected by RT-qPCR. (**B**) phosphorylation of NF-κB-p65 and expression of proteins NKRF, Bcl-2, CyclinD1 and VEGF in C-LCs of each group were detected by western blotting. The tests were carried out on three biological triplicates, and the data are expressed as the mean ± SD. ** *p* < 0.01

Based on the previous hypothesis, ICL can indirectly inhibits NF-κB activity and its leading LC progression by regulating NKRF expression through miR19-3p/NKRF pathway, we further verified the pathway of ICL in C-LCs. The RT-qPCR data showed that there was no significant difference of ICL levels in C-LCs between control and Lv-NC groups (*p* > 0.05, vs. control group), but ICL was significantly increased in C-LCs of Lv-ICL and Lv-shRNA-NKRF + Lv-ICL groups (*p* < 0.01 vs. control group), there was no significant difference of ICL levels in C-LCs between Lv-ICL and Lv-shRNA-NKRF + Lv-ICL groups (*p*>0.05). RT-qPCR data also showed that hsa-miR19-3p was the lowest in C-LCs of Lv-ICL group and highest in Lv-shRNA-NKRF + Lv-ICL groups (p < 0.05 vs. control group), and there was no significant difference of hsa-miR19-3p levels in C-LCs between control and Lv-NC groups (*p*>0.05) (Fig. 4A). Western blotting showed that the expression of NKRF was the highest in C-LCs of Lv-ICL group and lowest in C-LCs of Lv-shRNA-NKRF + Lv-ICL groups (*p* < 0.01 vs. control group), and there was no significant difference in NKRF protein expression of C-LCs between the control and Lv-NC groups (*p*>0.05) (Fig. 4B). The above results showed that ICL could affect the expression of hsa-miR19-3p and NKRF, while NKRF silencing could not affect ICL, suggesting that ICL is an independent factor affecting hsa-miR19-3p/NKRF. Furthermore, the results also showed that hsa-miR19-3p and NKRF showed a negative correlation in C-LCs of all groups, which suggested that hsa-miR19-3p and NKRF might be involved in an interactive regulatory pathway. A circular regulatory pathway hsa-miR19-3p/NKRF/NF-κB of seems to be able to explain the above phenomenon very well. The western blotting results also showed that the changes of NF-κB p65 phosphorylation in C-LCs of each group was completely opposite to that of NKRF expression. while the changes of tumor promoting factors Bcl-2, CyclinD1 and VEGF, downstream proteins regulated by NF-κB, were all consistent with phosphorylation of NF-κB p65 in C-LCs of each group (Fig. 4B).

### Exogenous ICL can effectively inhibit the growth of transplanted tumor of LC patient with COPD in PDX model and prolong the survival period of animals

**Fig. 5:**
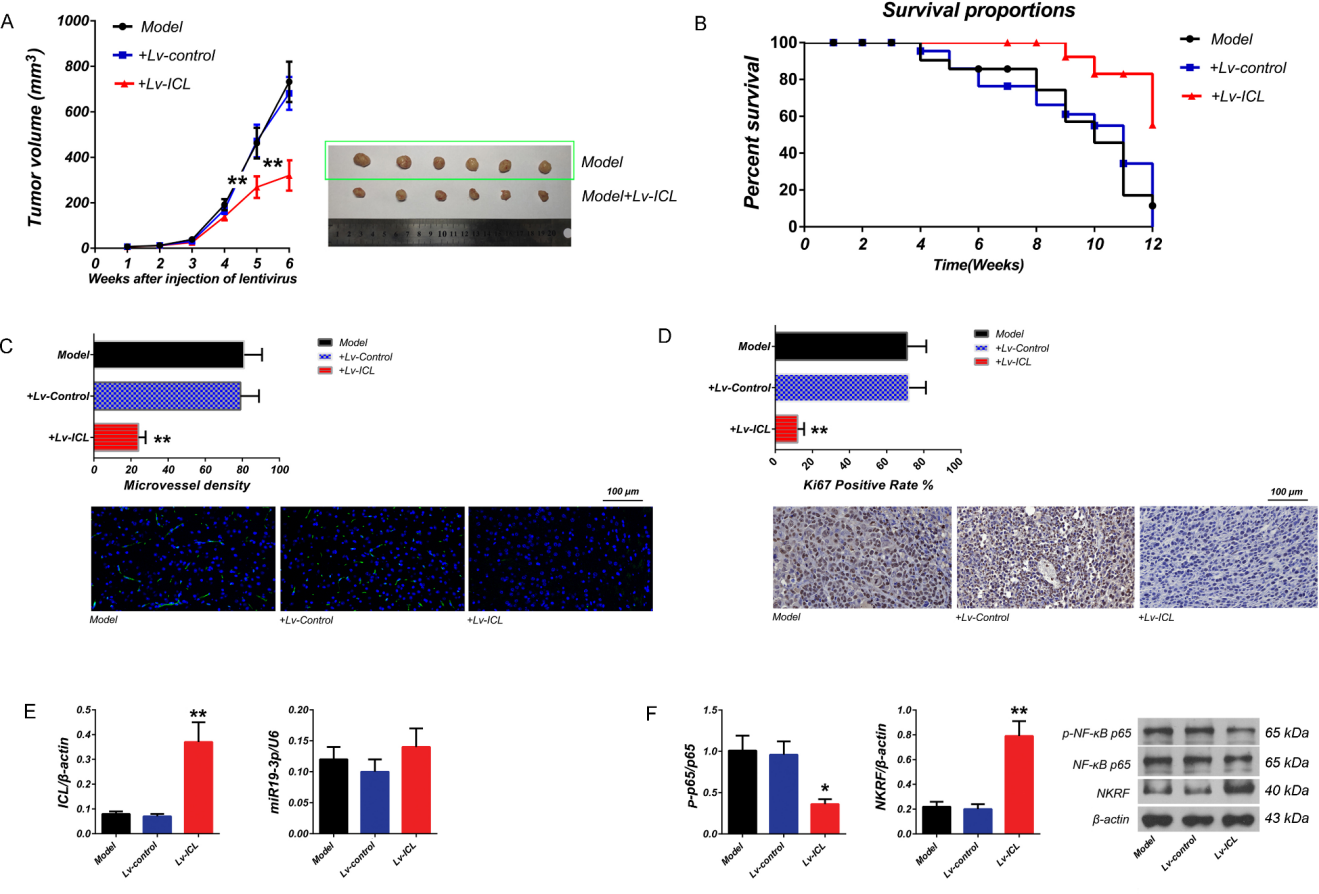
Analysis of the influence of ICL overexpression on growth of subcutaneous tumors and survival of tumor-bearing mice. (**A**) Tumor growth curve. The x-coordinate represents the injection time (weeks) and the y-coordinate represents the tumor volume (mm^3^). The number of animals in each group was six (n = 6). (**B**) Survival time of animals (n = 12). (**C**)The microvessel density in subcutaneous tumors was calculated. The immunostaining was visualized by fluorescence microscopy at 200× magnification. (**D**) The positive Ki67 assay. To analyze the Ki67 positive rate, the labeling index was performed. Three random images from every cell slide were photographed in triplicate. The labeling index was calculated by dividing the number of Ki67 positive cell nuclei by the total number of cells and multiplying by 100. (**E**) Detection of ICL and hsa-miR19-3p levels in subcutaneous tumor tissues by using RT-qPCR. (**F**) Detection of phosphorylation of NF-κB-p65 and protein expression of NKRF in subcutaneous tumor tissues by western blotting. Left, analysis of optical density. right: X-image of the target bands. Data are expressed as the means ± SD. ** *p* < 0.01, *p < 0.05

Six weeks after transplantation, the tumor volumes of subcutaneous tumors in the model, Lv-control and Lv-ICL groups were 731.93 ± 88.74, 681.18 ± 72.08 and 319.99 ± 66.65 mm^3^, respectively. Compared to the model group, the tumor inhibition rates in the Lv-control and Lv-ICL groups were 6.93% and 56.28% (*p* < 0.01 vs. model or Lv-control groups, Fig. 5A). Survival data of tumor-bearing mice showed that animals in the Lv-ICL group had the highest survival rate (*p* < 0.01 vs. model or Lv-control groups), there was no significant difference in mice between the model and Lv-control groups (*p*>0.05, Fig. 5B). Immunofluorescence staining of CD34 in the subcutaneous tumors showed that the density of nascent lymphatic vessels in the tumors of Lv-ICL group was significantly decreased than that of model or Lv-control groups (*p* < 0.01 vs. model or Lv-control groups), there was no significant difference between the model and Lv-control groups (*p*>0.05, Fig. 5C). Immunohistochemical staining showed that the changes in Ki67 positivity rates in the tumors from each group were consistent with the changes of CD34 protein (Fig. 5D). RT-qPCR showed that the expression of ICL was significantly enhanced in tumor tissues of Lv-ICL group than that of model and Lv-control groups (*p*<0.01,vs. model and Lv-control groups ), and there was no significant difference between the model and Lv-control groups (*p*>0.05). The expression of hsa-miR19-3p was enhanced in tumor tissues of Lv-ICL group, but there was no significant difference (*p*>0.05, vs. model and Lv-control groups), there was no significant difference between the model and Lv-control groups (*p*>0.05) (Fig. 5E). The results of western blotting showed that the expression of NKRF was significantly increased in tumor tissues of Lv-ICL group than that of model and Lv-control groups (*p*<0.01, vs. model and Lv-control groups ), there was no significant difference between the model and Lv-control groups (*p*>0.05). However, the phosphorylation of NF-κB-p65 was significantly decreased in tumor tissues of Lv-ICL group than that of model and Lv-control groups (*p*<0.01, vs. model and Lv-control groups ), and there was no significant difference between the model and Lv-control groups (*p*>0.05. Figure 5 F). Taken together, the in vivo data indicated that ICL can effectively inhibit the progression of subcutaneous tumors.

## Discussion

Studies have shown that at least 40% of LC patients had a history of COPD, and the mortality of LC patients with COPD is 2.6 times greater than that of those without COPD. According to statistics, the incidence rate of LC in COPD patients is as high as 800-1,700/100,000, which is 6–20 times greater than that of normal population [1, 2]. Therefore, the pathogenesis of LC in patients with COPD should have its own characteristics different from those of LC patients without COPD. Recent research shows that COPD is closely related to the incidence of LC, in terms of etiology, both of them can be caused by oxidative stress and occur in aging lungs. For example, as a contributing factor to the progression of LC and COPD, about 10^15^ free radicals, including reactive nitrogen and reactive oxygen species are contained by each cigarette. Oxidative stress can induce and maintain the oncogenic phenotype of cancer cells cause COPD by damaging DNA and inducing aberrant inflammation. In fact, that LC and COPD have the common genetic susceptibility is not only reflected in the pathological process caused by tobacco. Genome wide association analysis (GWAS) have found multiple common susceptibility sites using samples cohort from COPD and LC patients, indicating that some susceptibility loci used for determination of the susceptibility to COPD are also important for susceptibility to LC [17]. Therefore, it is believed that COPD and LC may actually be different aspects of the same disease, and COPD is a common and important driver of LC. Studies have shown that the presence of mild to moderate COPD could increase the risk of LC by three times within 10 years, and increase to 10 times in patients with severe COPD, which may be related to the abnormal inflammatory response of the lung in COPD patients. The inflammatory response of the lungs in COPD patients can induce mitotic disorder, aggravate the possibility of endogenous DNA damage into mutation, and cause proliferation in cancerous cells and tumor growth. The clinical and epidemiological survey data showed that chronic inflammation caused by airway obstruction also has a beneficial impact on LC incidence. It is gratifying that the new version of global initiative for chronic obstructive lung disease (GOLD) emphasizes that we should paid attention to the problem of COPD complicated with LC, and it was proposed that the early recognition of LC should be carry out to improve intervention effects on cancers of LC patients with COPD.

A number of studies have reported that the commonly used drugs of COPD, such as inhaled corticosteroids (ICS) and long-acting drugs β Receptor agonist (LABA), mainly the former, can not only effectively reduce various respiratory symptoms and acute exacerbation of COPD, but also can effectively prevent the occurrence of LC. A cohort study of 10,474 COPD patients with an average follow-up of 38 years showed that the risk of LC in COPD patients treated with drugs decreased in a dose-dependent manner compared to those without ICS, after adjusting for age, smoking history, smoking volume, history of other malignancies, basic diseases, bronchodilator application and other factors. The research also showed that the ICS + LABA combination is better than single drug in reducing the risk of LC in patients with COPD [18, 19]. Yu et al. studied the relationship between aspirin and LC related mortality in LC patients with COPD through a nationwide cohort. The study data showed that use of aspirin was associated with a 25% reduction in LC risk (SHR = 0.75, 95% confidence interval 0.65 to 0.87, *p* ≤ 0.001) and a 26% reduction in LC related mortality (SHR = 0.74, 95% confidence interval 0.64 to 0.86, *p* ≤ 0.001) in COPD patients. Therefore, the author believed that aspirin might be a potential drug for treatment of LC in patients with COPD [20]. Although the current retrospective study should not be regarded as sufficient evidence to include aspirin in the management strategy of COPD, the protective association between aspirin and LC deserves to be clarified by future randomized studies. All these studies have shown that the risk of LC must be highly valued in patients with COPD.

Studies have shown that the activation of nuclear transcription factor NF-κB is an important feature of pulmonary inflammatory diseases [21, 22]. The biological function of NF-κB is closely related to the pathophysiological processes such as immune response, inflammatory response, cell proliferation, transformation and apoptosis [23, 24]. NF-κB involved in all stages of inflammatory response and early immune response by regulating of TNF-α、IL-6、 IL-8、 IL-12、COX-2, adhesion molecules, chemokines and colony stimulating factors. In view of that activation of NF-κB participates in the pulmonary inflammatory reaction of COPD patients and maintains it for a long time, many researchers have believed that t NF-κB could be used as the primary target of COPD. Meanwhile, NF-κB has been proved to be involved in the regulation of multiple types of tumor progression [25]. NF-kB usually exists in the cytoplasm of almost all the types of cells in an inactive form, it can transfer from the cytoplasm to the nucleus and regulate the transcription of multiple downstream genes after being activated. NF kappa B repressing factor (NKRF) modulates transcription activity of NF-κB proteins through a direct interaction with Rel homology domain. Studies have shown that in most cases, there is a delicate balance between them, and maintain the normal biological function of NF-κB. In our study, the clinical tissues test data clearly showed that expression of NKRF was significantly decreased and phosphorylation of NF-κB -p65 significantly enhanced in LC tissues of LC patient with COPD than those without COPD. Correspondingly, there was no significant difference in the expression of NKRF mRNA between the two LC tissues. This suggests that the activation of NF-κB in LC progression of COPD patients may be due to the decreased of NKRF protein caused by the abnormal posttranscriptional regulation mechanism.

**Fig. 6:**
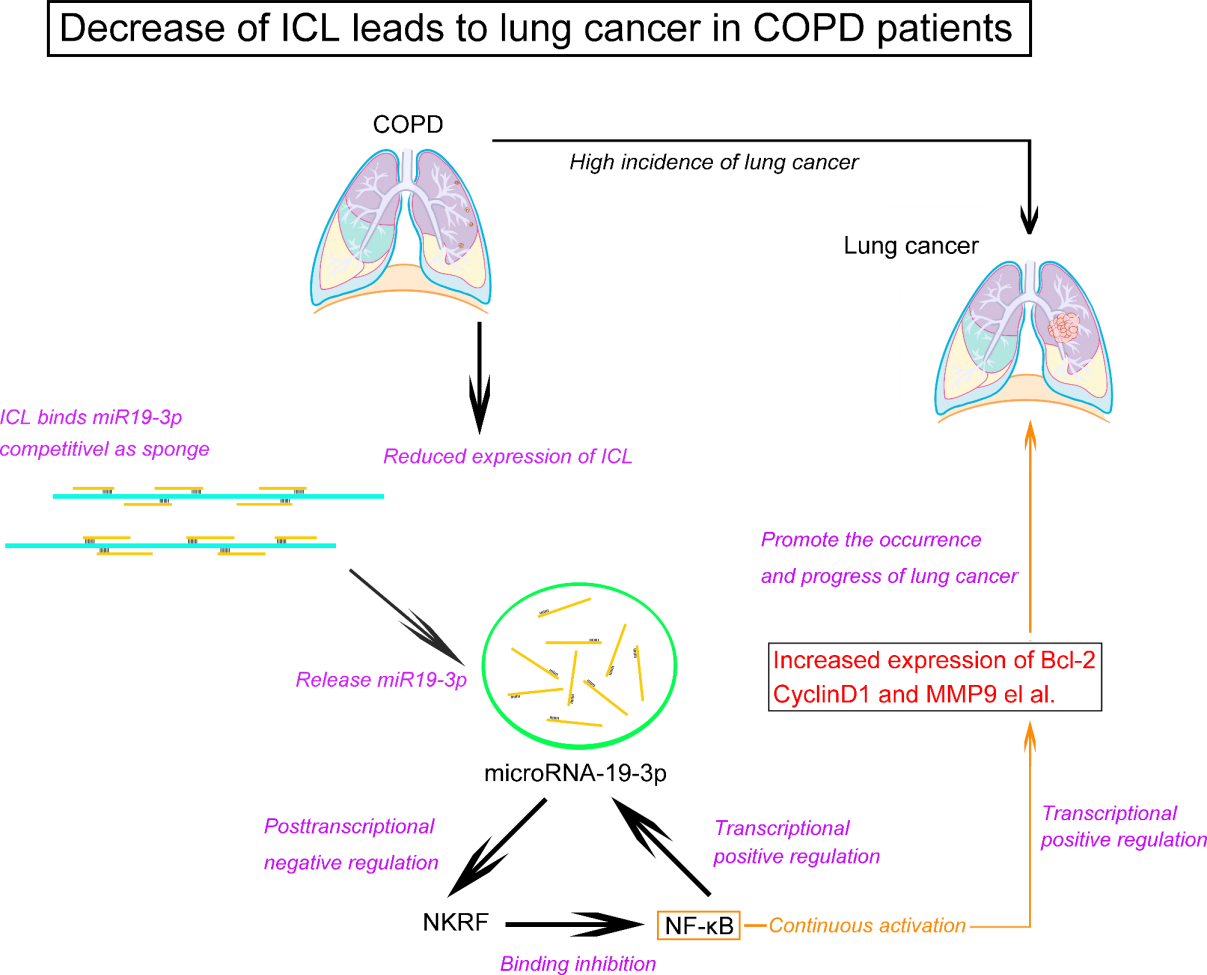
Schematic diagram of the mechanism of ICL participating in the regulation of the occurrence and progression of LC in patients with COPD. The right frame illustrates a continuously activated hsa-miR19-3p/NKRF/ NF-κB circular regulatory pathway which leads to the occurrence and progression of LC in COPD patients. The left frame illustrates a proposed model of ICL in inhibiting the occurrence and progression of LC in COPD patients by blocking hsa-miR19-3p/NKRF/NF- κ B pathway

As a classic post-transcriptional regulatory mechanism, we think there might be a miRNA which regulates NKRF at post-transcriptional level. We used the bioinformatics software “TargetScan and miRanda” to predict the miRNAs with theoretical binding sites on the 3’UTR of NKRF mRNA, and then verified these binding sites by luciferase method. The results showed that hsa-miR19-3p could negatively regulate the expression of NKRF protein by binding to its 3’UTR. However, the subsequent detection data and correlation analysis make us confused about our previous hypothesis. The expression of hsa-miR19-3p and NKRF protein only maintained a negative correlation in the LC tissues of LC patients with COPD. In other words, hsa-miR19-3p seems to have lost its function on inhibiting NKRF expression in LC tissues of LC patients without COPD, this is consistent with the detection data of NF-κB p65 phosphorylation in LC tissues of LC patients without COPD is significantly lower than that of LC patients with COPD. So far, we believe that hsa-miR19-3p can indirectly activate NF-κB in LC tissues of LC patients with COPD by negatively regulating the expression of the NKRF. Previous studies have shown that miR-19 has abnormal expression in many cancers, such as breast, lung, colorectal, thyroid, and so on [26]. In the field of LC research, miR-19 has been proved to regulate lung cancer stem cells (CSCs) traits and play a key role in SFN intervention of lung CSCs through miR-19/GSK3 beta/beta catenin axis [27]. Other studies have shown that miR-19 can regulate the proliferation and metastasis of LC cells through MXD1 and affect the prognosis of LC patients [28]. Aripova et al. have found that the expression level of free-circulating hsa-miR-19b-3p was decreased in the blood plasma of patients with BA and ACOS and increased in patients with COPD in a study on detection and validation of new microRNAs as biomarkers for chronic lung diseases [29]. These studies together show that miR-19 plays an important role in the pathological progression of COPD and LC in patients. From our test data, it is not difficult to find that hsa-miR19-3p can promote the occurrence and progression of LC in COPD patients, and the inactivation of hsa-miR19-3p may be an important reason why the LC incidence rate of COPD patients is higher than that of normals. In addition, based on the fact that the positive correlation between hsa-miR19-3p and NF-κB p65 phosphorylation simultaneously exists in the LC tissues of LC patients with or without COPD, we verified that NF-κB can bind to the hsa-miR19-3p promoter and positively regulate its transcription through luciferase and CHIP experiments. So far, we have every reason to believe that a circular pathway hsa-miR19-3p/NKRF/NF-κB is likely to maintain the continuous activation of NF-κB in LC tissues of LC patients with COPD (As shown in Fig. 6).

The comparative analysis showed that the phosphorylation of NF-κB p65 in the LC tissues of LC patients with COPD is significantly higher than that of those without COPD, suggesting that this circular regulatory pathway seems to be activated only in the LC tissues of LC patients with COPD. In sharp contrast, the operation of the circular pathway in LC tissues of the LC patients without COPD is blocked. Obviously, what leads to the activation and closure of the pathway should be the key link between the LC and COPD. We believe that there is likely to be a lncRNA which can weaken the regulatory function of hsa-miR19-3p on NKRF, this is in line with the inference and expectation theoretically. According to the theory, it is not difficult to imagine that this lncRNA should have two basic characteristics: [1] The expression of the lncRNA in LC tissues of LC patients with COPD is significantly lower than that of LC patients without COPD. [2] This lncRNA must have the structure of sponge of hsa-miR19-3p. It is reported that lncRNAs can competitively inhibit the binding of miRNA to its target gene [30]. Based on inferred characteristics, we screened and obtained a lncRNA-ICL which had the sponge structure of hsa-miR19-3p and was significantly decreases in LC tissues of LC patients with COPD compared to those without COPD. The luciferase experiments confirmed that ICL could significantly inhibit the binding of hsa-miR-19 to the 3’UTR of NKFB. Research data revealed that ICL was an independent influence factor of circular regulatory pathway hsa-miR19-3p/NKRF/NF-κB. Subsequent functional in vitro and in vivo analyses showed that overexpression of ICL could more effectively inhibit the proliferation, invasion and migration in primary tumor cells from LC patients with COPD than those without COPD, it also inhibited the growth in subcutaneous xenograft tumor of LC patients with COPD and prolong the survival of tumor-bearing mice. In addition, in the study of mechanism, we set up a response control through NKRF silence to evaluate the dominance of ICL’s pathway. At the same time, we collected LC tissues from LC patients with COPD and further established the patient-derived xenograft model (PDX) to verify whether ICL has the effect on inhibiting the progression of LC in COPD patients with the different genetic background.

The significance of this study is that we report for the first time that a circular pathway miR19-3p/NF-κB//NKRF participates in the occurrence and progression of LC in patients with COPD, and first revealed that ICL is an important reason for the high incidence of LC in patients with COPD. As shown in Fig. 6, our research confirms that the activation of circular pathway and continuous activation of NF-κB caused by the decrease of ICL in lung tissue and cells of COPD patients should promote carcinogenesis and tumor progression in COPD patients. This study provides a strong theoretical support for using ICL as a target for precise treatment and targeted therapy of LC in COPD patients. More importantly, we should not ignore the potential of ICL and its metabolites used as new clinical screening markers. It is undoubtedly encouraging that our ongoing research have shown that ICL can encode small molecular peptides which can be detected in BALF of COPD patients, we can imagine that if it can be stably detected in bronchoalveolar lavage fluid (BALF) and plasma, the ICL encoded polypeptide should have the potential to be used as non-invasive biomarkers for risk prediction and diagnosis of LC in COPD patients, this is consistent with the current research hotspots of lncRNAs. A study of Huang et al. indicates that the loss of HOXB-AS3 peptide is a critical oncogenic event in CRC metabolic reprogramming, and their findings uncover a complex regulatory mechanism of cancer metabolism reprogramming orchestrated by a peptide encoded by a lncRNA [31]. Pang et al. showed a new mechanism of HCC tumorigenesis promoted by ncRNA-encoded peptides, they also believed that the peptides can serve as a new target for HCC cancer therapy and a new biomarker for HCC diagnosis and prognosis [32]. Up to now, although the research on the biological functions of lncRNA and its encoded peptides has the broad prospects, there are also bottlenecks that restrict further research of them for example the development and establishment of new schemes suitable for clinical detection of lncRNAs and peptides and the the extended follow-up of patients, we undoubtedly believe that the prospect is bright.

## Conclusion

In conclusion, our study shows that the decreased of ICL promotes the occurrence and progress of LC in COPD patients by blocking circular pathway hsa-miR19-3p/NF-κB//NKRF. ICL has the potential to be used as a therapy target for LC in COPD patients, and its metabolites are expected to become a new marker for predicting the risk of LC among patients with COPD.

## Data Availability

The dataset supporting the conclusions of this article is included within the article.

## References

[1] Shireen Mirza RD, Clay MA, Koslow, Paul D, Scanlon. COPD Guidelines: A Review of the 2018 GOLD Report. Mayo Clin Proc. 2018;93(10):1488–1502. 10.1016/j.mayocp.2018.05.026 30286833

[2] Claus F, Vogelmeier GJ, Criner FJ, Martinez A, Anzueto, Peter J, Barnes J, Bourbeau (2017). Global strategy for the diagnosis, management, and Prevention of Chronic Obstructive Lung Disease 2017 Report. GOLD Executive Summary. Am J Respir Crit Care Med.

[3] Zhi XY, Zou XN, Hu M, Jiang Y, Jia MM, Yang GH (2015). Increased lung cancer mortality rates in the chinese population from 1973–1975 to 2004–2005: an adverse health effect from exposure to smoking. Cancer.

[4] Cecilia Mouronte-Roibás (2016). Virginia Leiro-Fernández, Alberto Fernández-Villar, Maribel Botana-Rial, Cristina Ramos-Hernández, Alberto Ruano-Ravina. COPD, emphysema and the onset of lung cancer. A systematic review. Cancer Lett.

[5] Juan P, de Torres JM, Marín C, Casanova C, Cote S, Carrizo (2011). Elizabeth Cordoba-Lanus. Lung cancer in patients with chronic obstructive pulmonary disease– incidence and predicting factors. Am J Respir Crit Care Med.

[6] Wang H, Yang L, Zou L, Huang D, Guo Y, Pan M (2012). Association between chronic obstructive pulmonary disease and lung cancer: a case-control study in Southern Chinese and a meta-analysis. PLoS ONE.

[7] Gu C, Li Y, Liu J, Ying X, Liu Y, Yan J (2017). LncRNA–mediated SIRT1/FoxO3a and SIRT1/p53 signaling pathways regulate type II alveolar epithelial cell senescence in patients with chronic obstructive pulmonary disease. Mol Med Rep.

[8] Tang W, Shen Z, Guo J, Sun S (2016). Screening of long non-coding RNA and TUG1 inhibits proliferation with TGF-β induction in patients with COPD. Int J Chron Obstruct Pulmon Dis.

[9] Li QN, Yang JZYX, Sun J, Zhou XM, Liu JF (2020). Transcriptome sequencing analysis of lncRNA expression in peripheral blood mononuclear cells from patients with COPD. J Hainan Med Univ.

[10] Raveh E, Matouk IJ, Gilon M, Hochberg A (2015). The H19 long non-coding RNA in cancer initiation, progression and metastasis - a proposed unifying theory. Mol Cancer.

[11] Liang Shi Z, Wang X, Geng Y, Zhang, Xue Z (2020). Exosomal miRNA-34 from cancer-associated fibroblasts inhibits growth and invasion of gastric cancer cells in vitro and in vivo. Aging.

[12] Deng X, Feng N, Zheng M, Ye X, Lin H, Yu X (2017). PM (2.5) exposure-induced autophagy is mediated by lncRNA loc146880 which also promotes the migration and invasion of lung cancer cells. Biochim Biophys Acta Gen Subj.

[13] Yang L, Wang H, Shen Q, Feng L, Jin H (2017). Long non-coding RNAs involved in autophagy regulation. Cell Death Dis.

[14] Ma J, Wu K, Liu K, Miao R (2018). Effects of MALAT1 on proliferation and apo- ptosis of human non-small cell lung cancer A549 cells in vitro and tumor xenograft growth in vivo by modulating autophagy. Cancer Biomark.

[15] Dunagin M, Cabili MN, Rinn J, Raj A (2015). Visualization of lncRNA by single-molecule fluorescence in situ hybridization. Methods Mol Biol.

[16] Angélica M, Herreño MJF, Rey L, Mejía JA, Alejandra Cañas OM, Moreno (2018). Primary lung cancer cell culture from transthoracic needle biopsy samples. Cogent Med.

[17] Global regional (2017). National deaths, prevalence, disability-adjusted life years, and years lived with disability for chronic obstructive pulmonary disease and asthma, 1990–2015: a systematic analysis for the global burden of Disease Study 2015. Lancet Respir Med.

[18] Aziz MIA, Tan LE, Wu DB, Pearce F, Chua GSW, Lin L (2018). Comparative efficacy of inhaled medications (ICS/LABA, LAMA, LAMA/LABA and SAMA) for COPD: a systematic review and network meta-analysis. Int J Chron Obstruct Pulmon Dis.

[19] Wang Y, Wen W, Yi Y, Zhang Z, Lubet RA, You M (2009). Preventive effects of bexarotene and budesonide in a genetically engineered mouse model of small cell lung cancer. Cancer Prev Res (Phila).

[20] Yu SY, Ip MS, Li X, Cheung KS, Ren QW, Wu MZ (2022). Low-dose aspirin and incidence of lung carcinoma in patients with chronic obstructive pulmonary disease in Hong Kong: a cohort study. PLoS Med.

[21] Remels AH, Gosker HR, Langen RC, Polkey M, Sliwinski P, Galdiz J (2014). Classical NF-κB activation impairs skeletal muscle oxidative phenotype by reducing IKK-α expression. Biochim Biophys Acta.

[22] Zaynagetdinov R, Sherrill TP, Gleaves LA, Hunt P, Han W, McLoed AG (2016). Chronic NF-κB activation links COPD and lung cancer through generation of an immunosuppressive microenvironment in the lungs. Oncotarget.

[23] Christman JW, Sadikot RT, Blackwell TS (2000). The role of nuclear factor-kappa B in pulmonary diseases. Chest.

[24] Binker MG, Binker-Cosen AA, Gaisano HY, Cosen-Binker LI (2008). Inhibition of Rac1 decreases the severity of pancreatitis and pancreatitis-associated lung injury in mice. Exp Physiol.

[25] Shishodia S, Aggarwal BB (2004). Nuclear factor-kappaB: a friend or a foe in cancer?. Biochem Pharmacol.

[26] Wang W, Zhang A, Hao Y, Wang G, Jia Z (2018). The emerging role of miR-19 in glioma. J Cell Mol Med.

[27] Zhu J, Wang S, Chen Y, Li X, Jiang Y, Yang X (2017). miR-19 targeting of GSK3β mediates sulforaphane suppression of lung cancer stem cells. J Nutr Biochem.

[28] Shao-hua LIN, LF ZHU, Xu-wei (2017). ZHANG Zhe-zhong. Research on protein MXD1 miR-19a regulating cell proliferation and metastasis of lung cancer. Chin J Health Lab Technol.

[29] Bersimbaev R, Aripova A, Bulgakova O, Kussainova А, Akparova A, Izzotti A (2021). The plasma levels of hsa-miR-19b-3p, hsa-miR-125b-5p, and hsamiR- 320c in patients with Asthma, COPD and Asthma-COPD Overlap Syndrome (ACOS). Microrna.

[30] Salmena L, Poliseno L, Tay Y, Kats L, Pandolfi PP (2011). A ceRNA hypothesis: the Rosetta Stone of a hidden RNA. language? Cell.

[31] Huang J-Z, Chen M, De Chen X-C, Gao S, Zhu H, Huang (2017). A peptide encoded by a putative lncRNA HOXB-AS3 suppresses Colon cancer growth. Mol Cell.

[32] Yanan Pang Z, Liu H, Han B, Wang W, Li C, Mao (2020). Peptide SMIM30 promotes HCC development by inducing SRC/YES1 membrane anchoring and MAPK pathway activation. J Hepatol.

